# Treatment outcomes among children younger than five years living with HIV in rural Zambia, 2008–2018: a cohort study

**DOI:** 10.1186/s12887-021-02793-y

**Published:** 2021-07-14

**Authors:** Jessica L. Schue, Janneke H. van Dijk, Francis Hamangaba, Mutinta Hamahuwa, Nkumbula Moyo, Philip E. Thuma, William J. Moss, Catherine G. Sutcliffe

**Affiliations:** 1 Johns Hopkins Bloomberg School of Public Health, 615 N. Wolfe St., Baltimore, MD USA; 2 Erasmus University, Burgemeester Oudlaan 50, 3062 PA Rotterdam, Netherlands; 3 Macha Research Trust, Choma, Zambia

**Keywords:** HIV, Pediatrics, Antiretroviral therapy, Sub-Saharan Africa

## Abstract

**Background:**

HIV testing and treatment guidelines for children in sub-Saharan Africa have evolved over time, such that children are now treated at younger ages. The objective of this study was to describe the treatment experience for immunologic, virologic, and growth outcomes among HIV-infected Zambian children younger than 5 years of age from 2008 to 2018.

**Methods:**

Participants enrolled in a clinical cohort study in Macha, Zambia and initiating antiretroviral treatment before 5 years of age between 2008 and 2015 were included in the analysis and followed up to the end of 2018. Outcomes, including growth, CD4+ T-cell percentage, viral suppression, and mortality, were evaluated among all children using longitudinal and survival analyses. Comparisons by age at treatment initiation (< 1, 1 to < 2, and 2 to < 5 years) were also evaluated.

**Results:**

Three hundred eighty-one children initiating treatment before 5 years of age between 2008 and 2015 were included in the analysis. Growth metrics and CD4+ T-cell percentage improved over time after treatment initiation. However, 20% of children remained underweight and 40% of children remained stunted after the first 36 months of treatment. 85% of children had a viral load < 400 copies/mL after 12 months of treatment. However, children < 1 year at treatment initiation were more likely to have a detectable viral load in the first 12 months of treatment and less likely to achieve viral suppression compared to older children. Mortality was highest in the first 12 months of treatment, among underweight children, and among children initiating treatment in 2008–2010 compared to 2011–2015.

**Conclusions:**

Most children initiating antiretroviral treatment from 2008 to 2015 in rural Zambia responded well to treatment. However, many children remained underweight and stunted, and experienced high mortality rates during the first few months of treatment. This supports continued efforts to improve early infant diagnosis, nutritional support, and pediatric drug formulations.

**Supplementary Information:**

The online version contains supplementary material available at 10.1186/s12887-021-02793-y.

## Background

Widespread adoption of universal treatment for all pregnant and breastfeeding women living with HIV has reduced the number of children acquiring perinatal HIV infection. Despite these improvements, globally an estimated 150,000 children 0–14 years of age were newly infected with HIV in 2019 and 1.8 million children 0–14 years of age were living with HIV, with approximately 90% residing in southern and eastern Africa [1].

Treatment guidelines for all children living with HIV have broadened over the past two decades. In the early part of this century, immunologic and disease severity criteria were required to initiate treatment [2]. With increasing recognition of the benefits of early treatment in preventing HIV-related morbidity and mortality, especially among children in the first months and years of life [3, 4], guidelines evolved to recommend initiating treatment as soon as possible after diagnosis [5]. These changing requirements have increasingly broadened the number of children eligible for treatment. At the same time, early infant diagnosis has increasingly become available, such that more countries are adopting birth testing for HIV exposed infants. This has resulted in children starting antiretroviral therapy (ART) at younger ages, with an increasing proportion starting ART before 2 years of age [6, 7]. In addition, first-line treatment regimens have evolved as new and more effective drugs have been developed and rolled out, including dosing formulations for infants [2, 5].

These changes in the profile of children initiating treatment may lead to changes in treatment outcomes as infants and young children have higher HIV viral loads and may start treatment prior to significant immunosuppression and disease progression [7–9]. The objective of this study was to describe the treatment experience in terms of immunologic, virologic, and growth outcomes among Zambian children initiating ART younger than 5 years of age from 2008 to 2018.

## Methods

### Study design and procedures

Participants in this analysis included the subset of children younger than 5 years of age who started ART and were enrolled in a pediatric HIV clinical cohort study at Macha Hospital in Southern Province, Zambia. The clinical cohort has been previously described [10]. Briefly, children testing positive for HIV during the study period were referred to the hospital from rural health centers within the catchment area. Early infant diagnosis of HIV became available in 2008 with recommended testing starting at 6 weeks of age, and then starting at birth in 2016 [11]. Guidelines for when to start ART in Zambia have expanded from treating only children with severe immunosuppression and disease progression in 2007 [12], to treating all children younger than 24 months in 2010 [13], and then all children younger than 15 years in 2013 [14] (Additional File 1 - Supplementary Table 1).

The clinical cohort study began enrollment in September 2007 and is ongoing. Children living with HIV and younger than 16 years of age were eligible. Written informed consent was obtained from parents or guardians and assent was obtained from children 8–15 years of age. Study visits occurred every 3 months and included a questionnaire and measurement of the child’s height and weight. Twice a year, a blood sample was collected, processed as plasma, and stored at -80 °C at Macha Research Trust for later testing to measure HIV viral load. During the period for this analysis, HIV viral load testing was performed at the Center for Infectious Disease Research Zambia (CIDRZ) lab in Lusaka, Zambia. At each study visit, data were abstracted from the child’s medical record to collect results of routine laboratory tests, including CD4+ T-cell count, CD4+ T-cell percentage, and hemoglobin. Children were classified as lost to follow-up if they missed at least two consecutive clinic visits over a six-month time period and were traced to determine their status. The study was approved by the Zambian Ministry of Health, the University of Zambia Biomedical Research Ethics Committee, and the Johns Hopkins Bloomberg School of Public Health Institutional Review Board.

### Statistical analysis

This analysis included all children starting ART younger than 5 years of age from January 1, 2008 to December 31, 2015. Outcomes during the first 3 years of treatment were considered to enable evaluation of both short and long-term outcomes with sufficient sample sizes. Children entered the analysis at ART initiation and exited at the first occurrence of death, 36 months after ART initiation, or their last visit prior to December 31, 2018. Children were considered lost to follow-up in the analysis if they had not been seen for at least 6 months prior to 36 months of ART or December 31, 2018 and were not known to have died or transferred to another clinic. Treatment outcomes were evaluated, including growth (measured by weight-for-age z-scores (WAZ) and height-for-age z-scores (HAZ) calculated based on the World Health Organization (WHO) growth standards [15]), CD4+ T-cell percentage, viral suppression, and mortality. Children with WAZ and HAZ scores below − 2 were defined as underweight and stunted, respectively. Severe immunodeficiency was defined by CD4+ T-cell percentage in accordance with the WHO 2006 guidelines [16]. Due to the decreasing lower limit of detection for HIV viral load testing that was reported over time, a threshold of < 400 copies per mL was used for the entire study period. A second threshold of < 1000 copies per mL also was evaluated, as this is commonly used in treatment guidelines in reference to treatment failure. Viral suppression was defined as two consecutive values < 400 copies per mL among available samples. Maintained viral suppression was defined as viral load < 400 copies/mL for all remaining samples tested after achieving viral suppression.

For all analyses, time was measured as time since ART initiation. The primary analysis evaluated outcomes among all children younger than 5 years of age. A secondary analysis was conducted comparing outcomes by age at ART initiation, categorized into three groups: < 1 year, 1 to < 2 years, and 2 to < 5 years.

For the WAZ, HAZ, and CD4+ T cell percentage outcomes, the analyses were restricted to children who had at least two study visits with non-missing data for that outcome between ART initiation and 36 months. For graphical displays of the data at specific time points, values were aggregated to within 45 days. Outcomes were evaluated using a mixed linear effects model with a random intercept and fixed slope. Models for WAZ and CD4+ T cell percentage included a spline at 7.5 months of age (the upper window of the 6-month measure). For comparisons by age group, models included an interaction term for months on ART and age group.

Viral load outcomes were evaluated in two ways. First, the proportion with viral load < 400 copies/mL was evaluated at each time point after ART initiation among children with at least one viral load measure available after ART initiation. Age groups were compared using logistic regression with generalized estimating equations, with an interaction term for month on ART and age group included in the model. Second, the proportion of children achieving viral suppression was evaluated among children with at least two viral load measures available after ART initiation. Among those achieving viral suppression, the proportion of children maintaining suppression was evaluated. The characteristics of children ever and never achieving viral suppression were compared using chi-square tests.

The mortality analysis included all children who initiated ART and was conducted as a survival analysis using Kaplan-Meier curves and Cox proportional hazards models.

For analyses comparing age groups, additional covariates, including sex, orphan status, stunting, underweight, severe immunodeficiency, ART era (2008–2010 vs 2011–2015), and ART regimen, that were known to be associated with the outcome or found to differ significantly between age groups at ART initiation were considered for inclusion in multivariable models. All analyses were conducted in Stata, version 16 (StataCorp LLC, College Station, Texas).

## Results

### Characteristics of study population

A total of 391 children younger than 5 years of age started ART between January 1, 2008 and December 31, 2015, of whom 381 were included in the analysis (10 children were excluded who enrolled in the study after being on ART for more than 3 years). At enrollment into the study, half (50.9%) the children were male and 12.6% were already receiving ART. Children initiated ART at a median age of 1.7 years (interquartile range [IQR]: 1.0, 2.8) and with a median CD4 T-cell percentage of 18.3% (IQR: 13.0, 24.2) (Table 1). The analysis included 86, 144, and 151 children initiating ART at < 1 year, 1 to < 2 years, and 2 to < 5 years of age, respectively (Table 1). Children who initiated ART at < 1 year of age were more likely to be male, to have been born to a mother who received antiretroviral drugs to prevent mother-to-child transmission, to have a parent as a primary caregiver, and to already be receiving ART at study enrollment compared to older children. At ART initiation, children < 1 year of age also had higher WAZ, lower hemoglobin levels, and were less likely to be stunted than older children. Children initiating ART at 2 to < 5 years of age had lower HIV viral loads than younger children, although this information was only available for 44% of participants.

**Table 1:** Characteristics of children younger than 5 years of age living with HIV in rural Zambia

	All ages		Age at ART initiation						
			< 1 year		1 to < 2 years		2 to < 5 years		
	N	Median (IQR)^**b**^	N	Median (IQR)^**b**^	N	Median (IQR)^**b**^	N	Median (IQR)^**b**^	***p***-value^**a**^
AT STUDY ENROLMENT									
Age in years	381	1.6 (1.0, 2.5)	86	0.6 (0.5, 0.8)	144	1.4 (1.2, 1.6)	151	2.8 (2.1, 3.7)	< 0.001
Male sex, n (%)	381	194 (50.9)	86	53 (61.6)	144	75 (52.1)	151	66 (43.7)	0.03
CD4^+^ T-cell %	314	19.6 (14.6, 26.2)	75	23.9 (14.6, 31.0)	116	19.1 (15.1, 24.6)	123	19.5 (13.2, 24.6)	0.23
Hemoglobin	329	9.3 (8.4, 10.1)	74	9.1 (8.2, 10.0)	118	9.3 (8.4, 10.0)	137	9.5 (8.8, 10.4)	0.33
Receiving ART^c^ n (%)	381	48 (12.6)	86	18 (20.9)	144	14 (9.7)	151	16 (10.6)	0.03
Mother received ART for PMTCT, n (%)	380	69 (18.2)	85	26 (30.6)	144	27 (18.8)	151	16 (10.6)	< 0.001
Parent’s vital, n (%) status	380		85		144		151		0.31
Both alive		348 (91.6)		82 (96.5)		131 (91.0)		135 (89.4)	
One died		31 (8.2)		3 (3.5)		13 (9.0)		15 (9.9)	
Both died		1 (0.3)		0 (0.0)		0 (0.0)		1 (0.7)	
Primary caregiver, n (%)	380		85		144		151		0.006
Parent		345 (90.8)		85 (100.0)		133 (92.4)		127 (84.1)	
Grandparent		22 (5.8)		0 (0.0)		7 (4.9)		15 (9.9)	
Aunt/Uncle		11 (2.9)		0 (0.0)		4 (2.8)		7 (4.6)	
Other		2 (0.5)		0 (0.0)		0 (0.0)		2 (1.3)	
Primary caregiver education, n (%)	372		85		141		146		0.43
None		19 (5.1)		3 (3.5)		10 (7.1)		6 (4.1)	
Primary		234 (62.9)		52 (61.2)		93 (66.0)		89 (61.0)	
Secondary		118 (31.7)		30 (35.3)		37 (26.2)		51 (34.9)	
Higher		1 (0.3)		0 (0.0)		1 (0.7)		0 (0.0)	
AT ART INITIATION									
Age in years	381	1.7 (1.0, 2.8)	86	0.7 (0.5, 0.8)	144	1.5 (1.3, 1.7)	151	3.0 (2.5, 4.1)	< 0.001
Year of ART initiation	381		86		144		151		0.55
2008–2010		189 (49.6)		40 (46.5)		69 (47.9)		80 (53.0)	
2011–2015		192 (50.4)		46 (53.5)		75 (52.1)		71 (47.0)	
WAZ^d^	362	−2.0 (−3.0, −0.9)	80	− 1.5 (− 2.6, − 0.1)	140	−2.2 (− 3.4, − 1.1)	142	−2.1 (− 2.8, − 1.1)	0.03
Underweight^e,^ n (%)		178 (49.2)		30 (37.5)		76 (54.3)		72 (50.7)	0.05
HAZ^d^	334	−3.1 (−4.2, − 2.0)	76	− 2.5 (− 4.4, − 1.6)	129	−3.3 (− 4.4, − 2.3)	129	−3.1 (− 4.0, − 2.2)	0.38
Stunted ^f,^ n (%)		252 (75.4)		49 (64.5)		101 (78.3)		102 (79.1)	0.04
CD4%^d^	329	18.3 (13.0, 24.2)	67	19.1 (14.8, 28.1)	128	18.6 (13.4, 24.2)	134	17.2 (12.1, 22.2)	0.29
Severe immune deficiency^g,^ n (%)		175 (53.2)		36 (53.7)		73 (57.0)		66 (49.3)	0.45
Hemoglobin^d^	337	9.4 (8.4, 10.4)	70	9.1 (8.2, 9.9)	129	9.5 (8.5, 10.3)	138	9.7 (8.8, 10.5)	0.03
Viral load^d,h^	168	5.8 (5.2, 5.9)	53	5.9 (5.2, 6.1)	54	5.9 (5.4, 5.9)	61	5.5 (5.1, 5.9)	0.01
ART regimen, n (%)	372		84		142		146		< 0.001
AZT/3TC/EFV		56 (15.1)		5 (6.0)		18 (12.7)		33 (22.6)	
AZT/3TC/NVP		82 (22.0)		24 (28.6)		31 (21.8)		27 (18.5)	
D4T/3TC/EFV		50 (13.4)		6 (7.1)		27 (19.0)		17 (11.6)	
D4T/3TC/NVP		104 (28.0)		29 (34.5)		30 (21.1)		45 (30.8)	
Other		80 (21.5)		20 (23.8)		36 (25.4)		24 (16.4)	

*3TC* Lamivudine, *ART* antiretroviral therapy, *AZT* Zidovudine, *D4T* Stavudine, *EFV* Efavirenz, *HAZ* height-for-age z-score, *IQR* interquartile range, *NVP* Nevirapine, *PMTCT* prevention of mother to child transmission, *WAZ* weight-for-age z-score

^a^ *p*-value comparing across age groups using a Pearson’s chi-squared test for categorical variables and a Kruskal Wallis test for continuous variables

^b^ Unless otherwise specified

^c^ Some children entered the study already receiving ART

^d^ Most recent value available within the 3 months prior to ART initiation

^e^ Underweight: Weight-for-age Z-score less than −2

^f^ Stunted: height-for-age Z-score less than − 2

^g^ Severe Immunodeficiency: Defined according to CD4+ T-cell percentage/count threshold by age (< 12 months: 25% or < 1500 cells/mm^3^; 12–35 months: < 20% or < 750 cells/mm^3^; 36–59 months: < 15% or < 350 cells/mm^3^) based on the 2006 WHO Guidelines [16]

^h^ Viral load in log_10_

### Growth: HAZ and WAZ

**Fig. 1:**
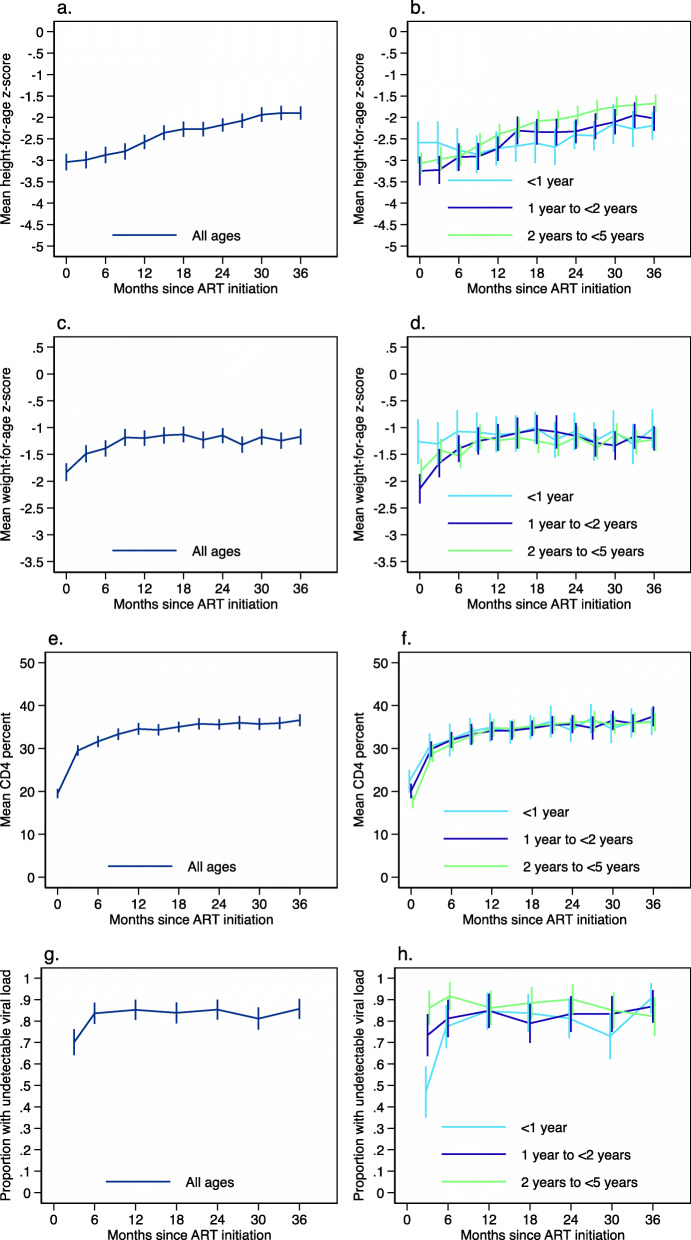
Growth, immunologic, and virologic outcomes among children living with HIV and receiving treatment in Zambia. **A** Height-for-age z-scores for all children < 5 years of age; **B** Height-for-age z-scores for children by age group (< 1 year, 1 to < 2 years, 2 to < 5 years); **C** Weight-for-age z-scores for all children < 5 years of age; **D** Weight-for-age z-scores for children by age group; **E** CD4+ T-cell percentage for all children < 5 years of age; **F** CD4+ T-cell percentage for children by age group; **G** Percentage with viral load < 400 copies/mL among all children < 5 years of age; **H** Percentage with viral load < 400 copies/mL for children by age group. Note: For reporting at each time point, values were aggregated to within 45 days; Error bars represent 95% confidence intervals

The analysis for HAZ included 352 children out of 353 children with at least two study visits. HAZ improved steadily after ART initiation, increasing a mean of 0.03 per month, such that the proportion with stunting decreased from 74.9% at ART initiation to 43.4% at 36 months on ART (Additional File 1 - Supplementary Table 2, Fig. 1A). HAZ improved after ART initiation among all age groups (Fig. 1A). Children < 1 year initiated ART with a higher HAZ compared to older children, although this difference was not statistically significant, and then had a significantly lower monthly increase in HAZ compared to children 2 to < 5 years of age, even after adjusting for underweight and severe immunodeficiency at ART initiation (Supplementary Table 5, Fig. 1B).

The analysis for WAZ included 353 children. WAZ improved over the first 6 months of ART, increasing a mean of 0.07 per month, and then stabilized (Fig. 1C), such that the proportion underweight decreased from 46.2% at ART initiation to 28.4% at 6 months on ART, and then remained at 20.4 to 26.9% until 36 months (Additional File 1 - Supplementary Table 3). Children initiating ART at 1 to < 2 years and 2 to < 5 years experienced the most improvement in WAZ. Children < 1 year initiated ART with higher WAZ compared to older children and experienced lower increases per month in the first 6 months on ART, such that all children reached and maintained similar z-scores until 36 months of ART (Additional File 1 - Supplementary Table 5, Fig. 1D).

### CD4+ T-cell percentage

The analysis for CD4+ T-cell percentage included 347 children. CD4+ T-cell percentage improved over the first 6 months of ART, increasing a mean of 1.6% per month, and then stabilized (Fig. 1E), such that the proportion with severe immunodeficiency decreased from 57.1% at ART initiation to 7.7% at 6 months on ART, and then remained at 0 to 6.6% until 36 months (Additional File 1 - Supplementary Table 4). Children 2 to < 5 years initiated ART with the lowest CD4+ T-cell percentage and experienced the most improvement in the first 6 months of ART. Children < 1 year and 1 to < 2 years initiated ART with higher CD4+ T-cell percentage compared to children 2 to < 5 years and experienced lower increases per month in the first 6 months on ART, such that all children reached and maintained similar levels by 36 months of ART (Additional File 1 - Supplementary Table 5, Fig. 1F).

### Viral load

The analysis for viral load included 237 children out of 356 children with at least one visit after ART initiation. Viral load decreased rapidly, such that 83% of children in care had a viral load < 400 copies/mL by 6 months of ART and this proportion remained stable through 36 months (Additional File 1 - Supplementary 6, Fig. 1G). Similar results were observed for a threshold of < 1000 copies per mL. Viral load decreased for all age groups. However, children initiating ART at < 1 year of age were less likely to have a viral load < 400 copies/mL at 3 and 6 months of ART compared to children initiating ART at 2 to < 5 years of age (Additional File 1 - Supplementary Table 7; Fig. 1H).

The analysis for viral suppression included 218 children out of 339 children with at least two visits after ART initiation, of whom 86% ever achieved viral suppression. Among children who ever achieved viral suppression and had at least one additional viral load measurement available, 83% maintained suppression for the remainder of their follow-up. There were no significant differences in the characteristics of children who ever and never achieved viral suppression (Additional File 1 - Supplementary Table 8). When comparing age groups, a smaller proportion of children initiating ART at < 1 year (76%) ever achieved viral suppression compared to children initiating ART at 1 to < 2 (88%) and 2 to < 5 (93%) years of age (*p* = 0.01) (Additional File 2 – Supplementary Figure 1). Among children who ever achieved viral suppression and had at least one additional viral load measure available, a similar proportion of children (< 1 year: 82%, 1 to < 2 years: 84%, and 2 to < 5 years: 84%) maintained suppression for the remainder of their follow-up. However, among children who did not maintain viral suppression, those initiating ART at < 1 year of age had fewer measures that were over 1000 copies per mL (27%) compared to children initiating ART at 1 to < 2 (53%) and 2 to < 5 (75%) years of age (*p* = 0.02).

### Survival

**Fig. 2:**
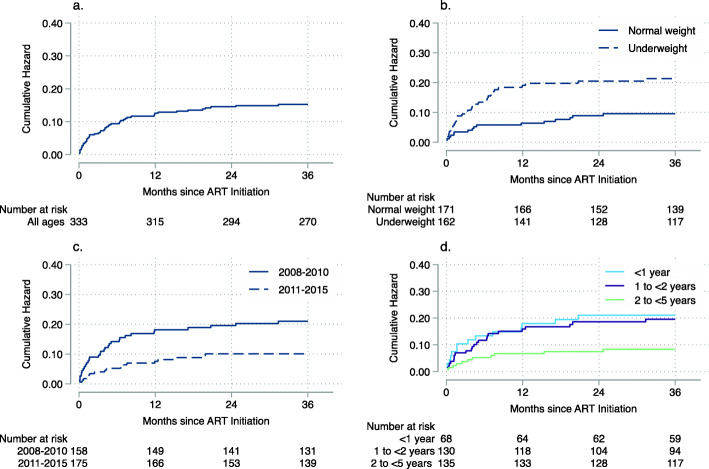
Mortality after treatment initiation among children living with HIV in rural Zambia, 2008–2018. **A** Mortality for all children < 5 years of age; **B** Mortality for children by underweight status (weight-for-age z-score < 2, weight-for-age z-score ≥ 2); **C** Mortality for children by ART era (2008–2010, 2011–2015); **D** Mortality for children by age group (< 1 year, 1 to < 2 years, 2 to < 5 years)

At the end of the study period, 279 (73.2%) children were alive and in care, 39 (10.2%) had transferred to another clinic, 49 (12.9%) had died, and 14 (3.7%) were lost to follow-up (Additional File 1 - Supplementary Table 9; also see Additional File 3 – Supplementary Figure 2 for further details on loss to follow-up). Over the first 36 months on ART, cumulative mortality was 15.2% (95% confidence interval [CI] 11.5, 20.2) and the mortality rate was 4.5 deaths per 1000 child-months (95% CI 3.4, 5.9). Most deaths were observed within the first 6–12 months of ART (Fig. 2A). Overall, children who were underweight at ART initiation (Fig. 2B) and who initiated ART in 2008–2010 (before guidelines changed to treat all children < 2 years [Supplementary Table 1]; compared to 2011–2015; Fig. 2C) had higher mortality. Mortality was lowest among children initiating ART at 2 to < 5 years of age and similar among children initiating ART at < 1 year and 1 to < 2 years of age (Fig. 2D; Additional File 1 – Supplemental Table 9). Risk factors for mortality were similar across age groups (Additional File 1 - Supplementary Table 10).

## Discussion

In this study in rural southern Zambia, children younger than 5 years of age initiating ART between 2008 and 2015 achieved good treatment outcomes, with improvements in growth and immunologic status within the first 6 months of ART. Most children achieved viral suppression within the first year of ART, although children initiating ART at < 1 year of age took longer than older children to achieve viral suppression. Mortality was highest in the first 6 months of ART, in the early years of the program, and among children who were underweight at ART initiation.

Growth metrics improved after initiating ART for all children in this study. The pattern of growth – with rapid gains in WAZ in the first 6 months of treatment and slower gains thereafter [17–19], and steady gains in HAZ throughout the first 36 months of treatment [18, 20] – was consistent with observations from other pediatric cohorts. Contrary to other cohorts [17, 19, 21], children initiating ART in the first year of life in this study tended to have higher WAZ and HAZ at ART initiation and lower rates of growth compared to older children. This may reflect differences in the characteristics of children (e.g., age distribution) included in studies and the services provided by treatment programs (e.g., nutritional support, which was intermittently available at the HIV clinic in Macha). While children in this study had improvements in WAZ and HAZ, a high proportion were underweight and stunted at ART initiation and after 3 years of ART. After 3 years of ART, levels of underweight (20.4%) and stunting (43.4%) remained higher than those observed among Zambian children overall and those residing in rural areas according to the Demographic and Health Survey conducted in 2018 (11.8 and 34.6%; 12.4 and 35.9%; respectively) [22].

While 83–86% of children in this study had a viral load < 400 copies/mL over 6–36 months of ART, 14% of children never achieved viral suppression. In addition, among those achieving viral suppression, 17% failed to maintain suppression for the duration of follow-up. This is consistent with other studies from sub-Saharan Africa, with only 36–78% of children achieving viral suppression [18, 23–26], although direct comparisons across studies are difficult due to differing limits of detection of assays and definitions of viral suppression. No factors associated with viral suppression were identified in this study. Importantly, this study found that infants took longer and were less likely to reach viral suppression compared to older children. This finding is consistent with a study among Kenyan infants and children [27], and may be due to a higher viral load prior to ART initiation or suboptimal pediatric dosing and adherence [28]. This has implications for monitoring treatment response and detecting virologic failure, as children initiating ART in infancy may need additional time before treatment failure can be determined and second line therapy considered. In addition, these findings highlight the continued need for drug development for infants so that simple and palatable regimens are available to ensure optimal adherence.

As found in other studies, the greatest risk of mortality occurred in the first 12 months of treatment [29, 30], among children who were underweight at ART initiation [18], and among infants [31–36]. Higher mortality among infants was expected due to a survival bias among older children, but biological factors may also have contributed, with more rapid disease progression and higher mortality among infants infected in utero or peri-partum [37], and suboptimal pediatric dosing and adherence in the younger age groups. There is strong evidence of the benefits of early treatment for children [3, 38, 39], and the continued high mortality observed among treated infants supports the need to facilitate early infant diagnosis and linkage to care so that infants can start ART as early as possible [40]. Encouragingly, the risk of mortality was lower in the period after guidelines were changed to initiate treatment in all children < 2 years of age [18], suggesting that these efforts are succeeding.

There are several limitations to this analysis. First, this was a clinical cohort and therefore data collected through routine clinical care were inconsistently available, which resulted in missing data. In addition, due to the difficulties in collecting blood from small children, an adequate study sample volume was not always available for viral load testing. This resulted in a larger proportion of children with missing viral load values prior to and after ART initiation who did not contribute to the analysis. As blood collection may be related to health status, the results may overestimate viral suppression among children in care. Second, this was an observational study conducted over a long period, during which changes were implemented in the timing of ART initiation and recommended and available ART regimens, which likely impacted the characteristics of children initiating ART and their treatment outcomes. To address this, the analyses included an evaluation of ART era and regimen. Third, this study was conducted at an HIV clinic in a rural area of Zambia, which may differ from other areas of Zambia or sub-Saharan Africa. While estimates of treatment response may differ, trends are likely to be consistent and so the findings add to the literature on treatment responses among infants and children in the region. Lastly, guidelines and ART regimens have continued to evolve since the conduct of this study, and therefore children currently initiating ART may experience different treatment outcomes than children in this study. However, the findings are still important to provide information on children currently receiving ART, to identify areas for continued monitoring of children initiating and receiving ART, and to provide a benchmark for treatment outcomes as regimens and monitoring tools improve.

## Conclusions

In conclusion, children initiating ART younger than 5 years of age in rural Zambia between 2008 and 2015 responded well to treatment. However, children starting ART as infants were less likely to achieve viral suppression than older children and experienced high mortality rates in the first few months of ART, supporting the need for continued improvements in early infant diagnosis and development of pediatric drug formulations. In addition, many children were underweight and stunted at ART initiation and remained so after 3 years of ART, putting them at increased risk for mortality. This highlights the need for nutritional programs for women and children living with HIV to ensure that they can achieve the best possible treatment outcomes.

## Supplementary Information

**Additional file 1: Supplementary Table 1.** Recommended pediatric treatment regimens in Zambia during the study period. **Supplementary Table 2.** Height-for-age z-scores after ART initiation, overall and stratified by age at ART initiation. **Supplementary Table 3.** Weight-for-age z scores after ART initiation, overall and stratified by age at ART initiation. **Supplementary Table 4.** CD4+ T-cell percentage after ART initiation, overall and stratified by age at ART initiation. **Supplementary Table 5.** Monthly change in height-for-age z-score, weight-for-age z-score, and CD4+ T-cell percent after ART initiation, by age at ART initiation. **Supplementary Table 6.** Proportion of children with HIV viral load < 400 and < 1000 copies/mL after ART initiation, overall and stratified by age at ART initiation. **Supplementary Table 7.** Comparison of viral load < 400 copies per mL after ART initiation (at 3, 6, and over 6 months on ART) by age group. **Supplementary Table 8.** Correlates of viral suppression among children living with HIV and receiving treatment in rural Zambia, overall and stratified by age at ART initiation. **9.** Outcomes among children living with HIV and receiving treatment in rural Zambia, overall and stratified by age at ART initiation. **10.** Risk factors for mortality among children living with HIV and receiving treatment in rural Zambia, overall and stratified by age at ART initiation.

**Additional file 2: Supplementary Figure 1.** Viral suppression among children living with HIV and receiving treatment in rural Zambia, 2008–2018. Note: Each row represents the experience of a participant. Viral suppression was defined as two consecutive viral loads < 400 copies per mL among available samples. (A)-(C): Children were grouped into 4 categories, each separated by a dashed line on the panel. Children in the 4th category never achieved viral suppression, the 3rd category reached suppression but did not maintain suppression, the 2nd category achieved and maintained viral suppression, and the 1st category reached suppression but did not have a follow up visit to measure maintenance. (A) The median (IQR) number of viral load measures available after ART initiation was 6 (5, 6). Forty-five of fifty-one children achieving viral suppression had at least one viral load measure available to assess maintenance of suppression. The median (IQR) number of additional viral load measures available was 2 (0, 3). (B) The median (IQR) number of viral load measures available after ART initiation was 6 (5, 6). Fifty-seven of sixty-nine children achieving viral suppression had at least one viral load measure available to assess maintenance of suppression. The median (IQR) number of additional viral load measures available was 2 (0, 4). (C) The median (IQR) number of viral load measures available after ART initiation was 5 (5, 6). Forty-five of fifty-one children achieving viral suppression had at least one viral load measure available to assess maintenance of suppression. The median (IQR) number of additional viral load measures available was 4 (3, 5).

**Additional file 3: Supplementary Figure 2.** Loss to follow-up after treatment initiation among children living with HIV in rural Zambia, 2008–2018.

## Data Availability

Under the Research Health Act, the Government of Zambia does not allow public access to data collected in Zambia. All investigators interested in the data are required to submit a written request to the Ministry of Health. Contact Dr. Catherine Sutcliffe (csutcli1@jhu.edu) to coordinate the request.

## Abbreviations

ART: Antiretroviral therapy
CI: Confidence interval
CIDRZ: Center for Infectious Disease Research in Zambia
HAZ: Height-for-age z-scores
HIV: Human immunodeficiency virus
IQR: Interquartile range
WAZ: Weight-for-age z-scores
WHO: World Health Organization
