# Supplementary material for: Treatment outcomes among children younger than five years living with HIV in rural Zambia, 2008–2018: a cohort study
Source: BMC Pediatr. 2021 Jul 14;21:315. doi: 10.1186/s12887-021-02793-y (PMC8278691; doi:10.1186/s12887-021-02793-y)
Supplement: Supplementary file 1 — Additional file 1: Supplementary Table 1. Recommended pediatric treatment regimens in Zambia during the study period. Supplementary Table 2. Height-for-age z-scores after ART initiation, overall and stratified by age at ART initiation. Supplementary Table 3. Weight-for-age z scores after ART initiation, overall and stratified by age at ART initiation. Supplementary Table 4. CD4+ T-cell percentage after ART initiation, overall and stratified by age at ART initiation. Supplementary Table 5. Monthly change in height-for-age z-score, weight-for-age z-score, and CD4+ T-cell percent after ART initiation, by age at ART initiation. Supplementary Table 6. Proportion of children with HIV viral load < 400 and < 1000 copies/mL after ART initiation, overall and stratified by age at ART initiation. Supplementary Table 7. Comparison of viral load < 400 copies per mL after ART initiation (at 3, 6, and over 6 months on ART) by age group. Supplementary Table 8. Correlates of viral suppression among children living with HIV and receiving treatment in rural Zambia, overall and stratified by age at ART initiation. 9. Outcomes among children living with HIV and receiving treatment in rural Zambia, overall and stratified by age at ART initiation. 10. Risk factors for mortality among children living with HIV and receiving treatment in rural Zambia, overall and stratified by age at ART initiation. [file 12887_2021_2793_MOESM1_ESM.docx]

**Supplementary Table 1. Recommended pediatric treatment regimens in Zambia during the study period**

| Year | Age group | Treatment eligibility | CD4 criteria for ART initiation | Preferred 1st line | Preferred 2nd line^a^ |
| --- | --- | --- | --- | --- | --- |
| 2007 | <15 years | WHO stage 4 or 3: treat all WHO stage 2 or 1: CD4 guided | Severe immunodeficiency defined as: CD4% <25% for ≤ 11 months  CD4% <20% for 12-35 months  CD4% <15% for ≥36 months | AZT + 3TC + NVP/ EFV OR  D4T + 3TC + NVP/ EFV OR  ABC + 3TC + NVP/ EFV | ddl + ABC + LPV-r or NFV OR  ddl + AZT + LPV-r or NFV |
| 2010 | <24 months | At HIV diagnosis | None | AZT + 3TC + NVP  (or LPV/r if previous NNRTI exposure) | ABC + 3TC + LPV/r |
| 2010 | ≥ 24 months | WHO stage 4 or 3: treat all WHO stage 2 or 1: CD4 guided | CD4% ≤25% for 24-59 months  CD4% ≤350 cells/mm^3^ for ≥5 years | AZT + 3TC + NVP  (or EFV if >3 years) | ABC + 3TC + LPV/r |
| 2013 | 6 weeks to < 3 months | At HIV diagnosis | None | AZT + 3TC + LPV/r (with switch to ABC at 3 months of age) | No switch. Improve adherence and refer to next level |
| 2013 | 3 months to < 5 years^b^ | At HIV diagnosis | None | ABC + 3TC + LPV/r | <3 years: No switch  ≥3 years and NNRTI naïve/non-exposed: ABC + 3TC + EFV |
| 2013 | 5 to <15 years | At HIV diagnosis | None | TDF + XTC + EFV | AZT + 3TC + LPV/r |
| 2016 | 0-2 weeks | At HIV diagnosis | None | AZT + 3TC + NVP | N/A |
| 2016 | 2 weeks to <5 years | At HIV diagnosis | None | ABC + 3TC + LPV/r | AZT + 3TC + RAL |
| 2016 | 5 to <10 years | At HIV diagnosis | None | ABC + 3TC + EFV | AZT + 3TC + LPV/r or ATV/r |
| 2016 | 10 to <15 years | At HIV diagnosis | None | TDF + XTC + EFV | AZT + 3TC + LPV/r or ATV-r |
| 2018 | 0-2 weeks | At HIV Diagnosis | None | AZT + 3TC + NVP | Consult physician |
| 2018 | 2 weeks to <10 years | At HIV Diagnosis | None | ABC + 3TC + LPV/r | AZT + 3TC + RAL |
| 2018 | 10 to <15 years | At HIV Diagnosis | None | TDF/TAF + XTC + DTG | AZT + 3TC + LPV/r or ATV/r |

^a^ Preferred 2^nd^ line provided for preferred 1^st^ line. Additional 2^nd^ line regimens are recommended for children failing on other 1^st^ line regimens.

^b^ All children <15 years of age eligible for cART at diagnosis

3TC: Lamivudine, ABC: Abacavir, ART: antiretroviral therapy, ATV/r: Atazanavir / Ritonavir, AZT: Zidovudine, cART: combination antiretroviral therapy, D4T: Stavudine, ddI: Didanosine, DTG: Dolutegravir, EFV: Efavirenz, LPV/r: Lopinavir / Ritonavir, NFV: Nelfinavir, NVP: Nevirapine, NNRTI: non-nucleoside reverse transcriptase inhibitor, RAL: Raltegravir, TDF: Tenofivir, XTC: Emtricitabine

**Supplementary Table 2. Height-for-age z-scores after ART initiation, overall and stratified by age at ART initiation**

| Month on ART | All ages | | | <1 year | | | 1 to <2 years | | | 2 to 5 years | | |
| --- | --- | --- | --- | --- | --- | --- | --- | --- | --- | --- | --- | --- |
|  | **n** | **mean (95% CI)** | **Stunted %** | **n** | **mean (95% CI)** | **Stunted %** | **n** | **mean (95% CI)** | **Stunted %** | **n** | **mean (95% CI)** | **Stunted %** |
| 0 | 291 | -3.04 (-3.23, -2.85) | 74.91 | 58 | -2.58 (-3.07, -2.10) | 58.62 | 113 | -3.25 (-3.59, -2.91) | 79.65 | 120 | -3.07 (-3.32, -2.81) | 78.33 |
| 3 | 271 | -2.99 (-3.19, -2.79) | 74.17 | 53 | -2.59 (-3.11, -2.07) | 58.49 | 103 | -3.22 (-3.55, -2.90) | 77.67 | 115 | -2.97 (-3.25, -2.69) | 78.26 |
| 6 | 266 | -2.88 (-3.06, -2.69) | 78.20 | 52 | -2.75 (-3.26, -2.24) | 69.23 | 100 | -2.92 (-3.24, -2.60) | 82.00 | 114 | -2.89 (-3.14, -2.64) | 78.95 |
| 9 | 254 | -2.79 (-2.98, -2.60) | 73.23 | 57 | -2.86 (-3.30, -2.41) | 70.18 | 96 | -2.91 (-3.22, -2.60) | 80.21 | 101 | -2.64 (-2.92, -2.37) | 68.32 |
| 12 | 254 | -2.57 (-2.74, -2.41) | 69.69 | 50 | -2.72 (-3.14, -2.31) | 68.00 | 90 | -2.73 (-3.02, -2.44) | 76.67 | 114 | -2.39 (-2.63, -2.15) | 64.91 |
| 15 | 243 | -2.36 (-2.53, -2.19) | 60.91 | 53 | -2.67 (-3.04, -2.29) | 64.15 | 79 | -2.31 (-2.63, -1.98) | 63.29 | 111 | -2.24 (-2.48, -2.00) | 57.66 |
| 18 | 241 | -2.27 (-2.45, -2.09) | 58.92 | 48 | -2.60 (-3.08, -2.12) | 64.58 | 82 | -2.34 (-2.64, -2.03) | 63.41 | 111 | -2.08 (-2.33, -1.84) | 53.15 |
| 21 | 233 | -2.27 (-2.44, -2.10) | 54.08 | 46 | -2.69 (-3.12, -2.26) | 56.52 | 76 | -2.34 (-2.66, -2.03) | 53.95 | 111 | -2.05 (-2.26, -1.84) | 53.15 |
| 24 | 227 | -2.18 (-2.33, -2.02) | 51.54 | 45 | -2.41 (-2.75, -2.06) | 62.22 | 81 | -2.32 (-2.60, -2.04) | 53.09 | 101 | -1.96 (-2.19, -1.73) | 45.54 |
| 27 | 203 | -2.08 (-2.25, -1.91) | 49.26 | 37 | -2.42 (-2.79, -2.06) | 62.16 | 77 | -2.21 (-2.51, -1.91) | 53.25 | 89 | -1.82 (-2.06, -1.58) | 40.45 |
| 30 | 216 | -1.94 (-2.11, -1.76) | 43.98 | 40 | -2.16 (-2.64, -1.67) | 60.00 | 70 | -2.10 (-2.40, -1.80) | 47.14 | 106 | -1.74 (-1.97, -1.52) | 35.85 |
| 33 | 216 | -1.90 (-2.07, -1.73) | 42.59 | 44 | -2.27 (-2.69, -1.84) | 52.27 | 69 | -1.95 (-2.26, -1.63) | 43.48 | 103 | -1.71 (-1.93, -1.49) | 37.86 |
| 36 | 198 | -1.90 (-2.06, -1.74) | 43.43 | 41 | -2.19 (-2.55, -1.84) | 56.10 | 65 | -2.02 (-2.32, -1.73) | 47.69 | 92 | -1.67 (-1.89, -1.46) | 34.78 |

ART: antiretroviral therapy, CI: confidence interval, Stunted: height-for-age Z-score less than -2

**Supplementary Table 3. Weight-for-age z scores after ART initiation, overall and stratified by age at ART initiation**

| Month on ART | All ages | | | <1 year | | | 1 to <2 years | | | 2 to 5 years | | |
| --- | --- | --- | --- | --- | --- | --- | --- | --- | --- | --- | --- | --- |
|  | **n** | **mean (95% CI)** | **Under-**  **weight %** | **n** | **mean (95% CI)** | **Under-**  **weight %** | **n** | **mean (95% CI)** | **Under-weight %** | **n** | **mean (95% CI)** | **Under-weight %** |
| 0 | 305 | -1.83 (-2.00, -1.67) | 46.23 | 58 | -1.26 (-1.68, -0.84) | 34.48 | 120 | -2.14 (-2.41, -1.87) | 51.67 | 127 | -1.80 (-2.02, -1.58) | 46.46 |
| 3 | 280 | -1.49 (-1.64, -1.33) | 30.71 | 56 | -1.30 (-1.71, -0.90) | 30.36 | 106 | -1.66 (-1.92, -1.40) | 36.79 | 118 | -1.42 (-1.63, -1.21) | 25.42 |
| 6 | 271 | -1.39 (-1.54, -1.24) | 28.41 | 54 | -1.07 (-1.47, -0.68) | 18.52 | 101 | -1.39 (-1.64, -1.14) | 26.73 | 116 | -1.53 (-1.74, -1.32) | 34.48 |
| 9 | 260 | -1.18 (-1.34, -1.03) | 26.92 | 58 | -1.08 (-1.45, -0.72) | 27.59 | 98 | -1.25 (-1.50, -1.00) | 27.55 | 104 | -1.18 (-1.41, -0.95) | 25.96 |
| 12 | 263 | -1.20 (-1.34, -1.06) | 20.91 | 52 | -1.13 (-1.47, -0.79) | 19.23 | 95 | -1.18 (-1.43, -0.93) | 21.05 | 116 | -1.24 (-1.44, -1.04) | 21.55 |
| 15 | 249 | -1.14 (-1.29, -1.00) | 22.89 | 55 | -1.11 (-1.45, -0.77) | 25.45 | 81 | -1.10 (-1.39, -0.81) | 25.93 | 113 | -1.19 (-1.39, -0.99) | 19.47 |
| 18 | 250 | -1.13 (-1.27, -0.98) | 23.20 | 50 | -1.00 (-1.30, -0.71) | 18 | 87 | -1.04 (-1.30, -0.77) | 20.69 | 113 | -1.25 (-1.47, -1.03) | 27.43 |
| 21 | 240 | -1.23 (-1.38, -1.08) | 25.00 | 46 | -1.23 (-1.57, -0.89) | 23.91 | 80 | -1.08 (-1.39, -0.77) | 25 | 114 | -1.33 (-1.53, -1.13) | 25.44 |
| 24 | 241 | -1.15 (-1.28, -1.01) | 23.24 | 48 | -1.07 (-1.43, -0.72) | 22.92 | 86 | -1.16 (-1.40, -0.92) | 25.58 | 107 | -1.17 (-1.36, -0.98) | 21.5 |
| 27 | 216 | -1.32 (-1.47, -1.17) | 25.46 | 42 | -1.26 (-1.62, -0.90) | 33.33 | 78 | -1.28 (-1.54, -1.03) | 26.92 | 96 | -1.37 (-1.59, -1.16) | 20.83 |
| 30 | 226 | -1.18 (-1.32, -1.03) | 18.58 | 43 | -1.06 (-1.45, -0.67) | 23.26 | 73 | -1.33 (-1.60, -1.07) | 21.92 | 110 | -1.11 (-1.30, -0.92) | 14.55 |
| 33 | 229 | -1.24 (-1.39, -1.10) | 24.45 | 45 | -1.31 (-1.68, -0.94) | 33.33 | 75 | -1.16 (-1.39, -0.93) | 21.33 | 109 | -1.27 (-1.50, -1.05) | 22.94 |
| 36 | 211 | -1.17 (-1.32, -1.02) | 20.38 | 46 | -1.02 (-1.39, -0.66) | 21.74 | 68 | -1.20 (-1.43, -0.98) | 23.53 | 97 | -1.22 (-1.44, -1.00) | 17.53 |

ART: antiretroviral therapy, CI: confidence interval, Underweight: Weight-for-age Z-score less than -2

**Supplementary Table 4. CD4+ T-cell percentage after ART initiation, overall and stratified by age at ART initiation**

| Month on ART |  | All ages |  | <1 year | | | 1 to <2 years | | | 2 to 5 years | | |
| --- | --- | --- | --- | --- | --- | --- | --- | --- | --- | --- | --- | --- |
|  | **n** | **mean (95% CI)** | **Severely immune deficient %** | **n** | **mean**  **(95% CI)** | **Severely immune deficient %** | **n** | **mean**  **(95% CI)** | **Severely immune deficient %** | **N** | **mean**  **(95% CI)** | **Severely immune deficient %** |
| 0 | 280 | 19.49 (18.44, 20.54) | 57.09 | 57 | 22.34 (19.65, 25.03) | 59.65 | 107 | 20.07 (18.40, 21.74) | 59.41 | 116 | 17.56 (16.07, 19.05) | 53.64 |
| 3 | 247 | 29.52 (28.30, 30.75) | 14.17 | 49 | 30.38 (27.27, 33.49) | 26.53 | 91 | 29.85 (28.07, 31.62) | 12.09 | 107 | 28.86 (26.89, 30.82) | 10.28 |
| 6 | 234 | 31.64 (30.37, 32.90) | 7.69 | 50 | 31.96 (28.14, 35.79) | 16.00 | 87 | 31.98 (30.13, 33.82) | 6.9 | 97 | 31.16 (29.44, 32.89) | 4.12 |
| 9 | 211 | 33.33 (32.00, 34.66) | 6.64 | 47 | 33.94 (30.72, 37.15) | 10.64 | 72 | 33.28 (30.87, 35.69) | 9.72 | 92 | 33.05 (31.23, 34.88) | 2.17 |
| 12 | 215 | 34.56 (33.22, 35.90) | 4.65 | 45 | 34.87 (31.55, 38.19) | 6.67 | 79 | 34.10 (31.95, 36.25) | 8.86 | 91 | 34.81 (32.77, 36.85) | 0 |
| 15 | 183 | 34.32 (33.02, 35.62) | 2.19 | 41 | 33.89 (31.16, 36.62) | 7.32 | 61 | 34.19 (32.02, 36.36) | 1.64 | 81 | 34.64 (32.56, 36.72) | 0 |
| 18 | 226 | 35.03 (33.82, 36.23) | 2.21 | 47 | 34.96 (32.12, 37.80) | 6.38 | 81 | 34.72 (32.91, 36.53) | 2.47 | 98 | 35.31 (33.35, 37.28) | 0 |
| 21 | 179 | 35.74 (34.43, 37.06) | 1.12 | 37 | 36.25 (32.48, 40.01) | 5.41 | 57 | 35.48 (33.44, 37.52) | 0 | 85 | 35.70 (33.85, 37.55) | 0 |
| 24 | 189 | 35.59 (34.38, 36.80) | 1.59 | 38 | 34.32 (31.48, 37.15) | 5.26 | 61 | 35.65 (33.53, 37.77) | 1.64 | 90 | 36.09 (34.30, 37.87) | 0 |
| 27 | 151 | 36.00 (34.44, 37.57) | 3.31 | 35 | 36.95 (33.52, 40.38) | 5.71 | 46 | 34.75 (32.06, 37.45) | 4.35 | 70 | 36.35 (33.96, 38.75) | 1.43 |
| 30 | 180 | 35.71 (34.36, 37.05) | 2.22 | 33 | 34.67 (31.16, 38.17) | 6.06 | 60 | 36.57 (34.33, 38.81) | 1.67 | 87 | 35.50 (33.53, 37.47) | 1.15 |
| 33 | 156 | 35.91 (34.48, 37.34) | 0.64 | 33 | 35.95 (32.51, 39.39) | 0 | 52 | 35.85 (33.79, 37.91) | 0 | 71 | 35.94 (33.61, 38.28) | 1.41 |
| 36 | 159 | 36.59 (35.21, 37.96) | 0 | 35 | 36.28 (33.08, 39.49) | 0 | 48 | 37.47 (35.16, 39.79) | 0 | 76 | 36.16 (34.08, 38.25) | 0 |

ART: antiretroviral therapy, CI: confidence interval, Severe Immunodeficiency: Defined by 2007 WHO Guidelines [19]

**Supplementary Table 5: Monthly change in height-for-age z-score, weight-for-age z-score, and CD4+ T-cell percent after ART initiation, by age at ART initiation**

|  | Crude | | | Adjusted^1^ | | |
| --- | --- | --- | --- | --- | --- | --- |
|  | **<1 year** | **1 to <2 years** | **2 to <5 years** | **<1 year** | **1 to <2 years** | **2 to <5 years** |
|  | **Mean (95% CI)** | **Mean (95% CI)** | **Mean (95% CI)** | **Mean (95% CI)** | **Mean (95% CI)** | **Mean (95% CI)** |
| Height-for-age z-score |  |  |  |  |  |  |
| Mean: at ART initiation | -2.76 (-3.04, -2.47) | -3.23 (-3.45, -3.01) | -3.04 (-3.25, -2.83) | -2.20 (-2.51, -1.88) | -2.47 (-2.73, -2.21) | -2.27 (-2.52, -2.02) |
| Mean: monthly change | 0 (0, 0.01) | 0.03 (0.03, 0.04) | 0.04 (0.04, 0.04) | 0 (0, 0.01) | 0.03 (0.03, 0.04) | 0.04 (0.04, 0.04) |
| Difference: at ART initiation | 0.28 (-0.07, 0.64) | -0.18 (-0.49, 0.12) | Ref | 0.08 (-0.26, 0.42) | -0.20 (-0.47, 0.08) | Ref |
| Difference: monthly change | -0.04 (-0.04, -0.03) | -0.01 (-0.01, 0) | Ref | -0.04 (-0.04, -0.03) | -0.01(-0.01, 0) | Ref |
|  |  |  |  |  |  |  |
| Weight-for-age z-score |  |  |  |  |  |  |
| Mean: at ART initiation | -1.25 (-1.51, -1.00) | -1.98 (-2.17, -1.79) | -1.82 (-2.00, -1.64) | -1.15 (-1.45, -0.85) | -1.83 (-2.06, -1.6) | -1.73 (-1.95, -1.51) |
| Mean: monthly change (0-6m) | 0.01 (-0.02, 0.04) | 0.09 (0.07, 0.11) | 0.07 (0.05, 0.09) | 0 (-0.03, 0.03) | 0.09 (0.07, 0.11) | 0.07 (0.05, 0.09) |
| Mean: monthly change (>6 m) | -0.01 (-0.02, 0) | 0 (-0.01, 0.01) | 0 (0, 0.01) | -0.01 (-0.02, 0) | 0 (0, 0.01) | 0 (0, 0.01) |
| Difference: at ART initiation | 0.57 (0.26, 0.88) | -0.16 (-0.43, 0.10) | Ref | 0.58 (0.24, 0.92) | -0.10 (-0.37, 0.18) | Ref |
| Difference: monthly change (0-6m) | -0.06 (-0.10, -0.03) | 0.02 (-0.01, 0.05) | Ref | -0.07 (-0.11, -0.04) | 0.01 (-0.01, 0.04) | Ref |
| Difference: monthly change (>6m) | -0.01 (-0.02, 0) | 0 (-0.01, 0.01) | Ref | -0.01 (-0.02, 0) | 0 (-0.01, 0.01) | Ref |
|  |  |  |  |  |  |  |
| CD4+ T-cell percent |  |  |  |  |  |  |
| Mean: at ART initiation | 23.47 (21.17, 25.76) | 22.42 (20.84, 24.01) | 20.24 (18.70, 21.78) | 25.05 (22.31, 27.79) | 23.75 (21.30, 26.20) | 21.75 (19.37, 24.12) |
| Mean: monthly change (0-6m) | 1.24 (0.99, 1.49) | 1.58 (1.41, 1.75) | 1.85 (1.69, 2.01) | 1.25 (1.02, 1.48) | 1.54 (1.37, 1.71) | 1.89 (1.73, 2.05) |
| Mean: monthly change (>6m) | 0.1 (0.02, 0.17) | 0.08 (0.03, 0.13) | 0.09 (0.04, 0.13) | 0.07 (0.00, 0.13) | 0.09 (0.04, 0.14) | 0.07 (0.03, 0.13) |
| Difference: at ART initiation | 3.23 (0.47, 5.99) | 2.18 (-0.03, 4.39) | Ref | 3.30 (0.66, 5.94) | 2.00 (-0.21, 4.21) | Ref |
| Difference: monthly change (0-6m) | -0.61 (-0.90, - 0.31) | -0.27 (-0.50, -0.04) | Ref | -0.64 (-0.92, -0.37) | -0.35 (-0.58, -0.12) | Ref |
| Difference: monthly change (>6m) | 0.01 (-0.08, 0.09) | -0.01 (-0.08, 0.06) | Ref | -0.01 (-0.08, 0.07) | 0.02 (-0.05, 0.08) | Ref |

ART: antiretroviral therapy, CI: confidence interval, Ref: reference group

^1^Height-for-age model was adjusted for underweight and severe immunodeficiency at ART initiation; Weight-for-age model was adjusted for severe immunodeficiency at ART initiation; CD4+ T-cell percent model was adjusted for sex and stunted at ART initiation

**Supplementary Table 6. Proportion of children with HIV viral load <400 and <1000 copies/mL after ART initiation, overall and stratified by age at ART initiation**

| Month  on ART | All ages | | <1 year | | 1 to <2 years | | 2 to 5 years | |
| --- | --- | --- | --- | --- | --- | --- | --- | --- |
|  | **n** | **Proportion (95% CI)** | **n** | **Proportion (95% CI)** | **n** | **Proportion (95% CI)** | **n** | **Proportion (95% CI)** |
| Viral load <400 copies/mL | | |  | |  |  |  |  |
| 3 | 171 | 0.70 (0.63, 0.77) | 49 | 0.47 (0.32, 0.61) | 64 | 0.73 (0.62, 1) | 58 | 0.86 (0.77, 0.95) |
| 6 | 180 | 0.83 (0.77, 0.88) | 55 | 0.75 (0.63, 0.86) | 64 | 0.81 (0.71, 1) | 61 | 0.92 (0.85, 0.99) |
| 12 | 186 | 0.85 (0.80, 0.90) | 52 | 0.85 (0.74, 0.95) | 66 | 0.85 (0.76, 1) | 68 | 0.85 (0.77, 0.94) |
| 18 | 163 | 0.84 (0.79, 0.90) | 43 | 0.84 (0.72, 0.95) | 58 | 0.79 (0.69, 1) | 62 | 0.89 (0.81, 0.97) |
| 24 | 158 | 0.85 (0.79, 0.90) | 49 | 0.80 (0.68, 0.91) | 48 | 0.83 (0.72, 0.94) | 61 | 0.90 (0.83, 0.98) |
| 30 | 138 | 0.81 (0.75, 0.88) | 37 | 0.73 (0.58, 0.88) | 48 | 0.83 (0.72, 0.94) | 53 | 0.85 (0.75, 0.95) |
| 36 | 126 | 0.86 (0.80, 0.92) | 32 | 0.91 (0.80, 1.01) | 38 | 0.87 (0.76, 0.98) | 56 | 0.82 (0.72, 0.92) |
| Viral load <1000 copies/mL | | |  | |  |  |  |  |
| 3 | 171 | 0.80 (0.73, 0.86) | 49 | 0.57 (0.43, 0.72) | 64 | 0.88 (0.79, 0.96) | 58 | 0.90 (0.82, 0.98) |
| 6 | 180 | 0.84 (0.79, 0.89) | 55 | 0.78 (0.67, 0.89) | 64 | 0.83 (0.73, 0.92) | 61 | 0.92 (0.85, 0.99) |
| 12 | 186 | 0.85 (0.80, 0.91) | 52 | 0.85 (0.74, 0.95) | 66 | 0.86 (0.78, 0.95) | 68 | 0.85 (0.77, 0.94) |
| 18 | 163 | 0.86 (0.80, 0.91) | 43 | 0.86 (0.75, 0.97) | 58 | 0.83 (0.73, 0.93) | 62 | 0.89 (0.81, 0.97) |
| 24 | 158 | 0.87 (0.82, 0.93) | 49 | 0.86 (0.76, 0.96) | 48 | 0.85 (0.75, 0.96) | 61 | 0.90 (0.83, 0.98) |
| 30 | 138 | 0.84 (0.78, 0.90) | 37 | 0.76 (0.61, 0.90) | 48 | 0.88 (0.78, 0.97) | 53 | 0.87 (0.77, 0.96) |
| 36 | 126 | 0.88 (0.82, 0.94) | 32 | 0.94 (0.85, 1) | 38 | 0.89 (0.79, 1) | 56 | 0.84 (0.74, 0.94) |

ART: antiretroviral therapy; CI: confidence interval

**Supplementary Table 7. Comparison of viral load <400 copies per mL after ART initiation (at 3, 6, and over 6 months on ART) by age group**

| Months since ART initiation | Crude | | | Adjusted^1^ | | |
| --- | --- | --- | --- | --- | --- | --- |
|  | **<1 year** | **1 to <2 years** | **2 to < 5 years** | **<1 year** | **1 to <2 years** | **2 to <5 years** |
|  | **OR (95% CI)** | **OR (95% CI)** | **OR (95% CI)** | **OR (95% CI)** | **OR (95% CI)** | **OR (95% CI)** |
| 3 months | 0.12 (0.05, 0.30) | 0.40 (0.16, 0.96) | Ref | 0.10 (0.04, 0.27) | 0.33 (0.12, 0.90) | Ref |
| 6 months | 0.39 (0.16, 0.95) | 0.69 (0.27, 1.75) | Ref | 0.30 (0.11, 0.85) | 0.66 (0.23, 1.83) | Ref |
| > 6 months | 0.64 (0.28, 1.49) | 0.77 (0.33, 1.78) | Ref | 0.58 (0.21, 1.59) | 0.65 (0.25, 1.72) | Ref |

ART: antiretroviral therapy; CI: confidence interval; OR: odds ratio; Ref: reference group

^1^ Adjusted for sex and severe immunodeficiency at ART initiation

**Supplementary Table 8. Correlates of viral suppression among children living with HIV and receiving treatment in rural Zambia, overall and stratified by age at ART initiation**

|  | All ages | | | <1 year | | | 1 to <2 years | | | 2 to <5 years | | |
| --- | --- | --- | --- | --- | --- | --- | --- | --- | --- | --- | --- | --- |
|  | **Ever**  **n (%)** | **Never**  **n (%)** | **p-value^1^** | **Ever**  **n (%)** | **Never**  **n (%)** | **p-value^1^** | **Ever**  **n (%)** | **Never**  **n (%)** | **p-value^1^** | **Ever**  **n (%)** | **Never**  **n (%)** | **p-value^1^** |
| N | 188 | 30 |  | 51 | 16 |  | 69 | 9 |  | 68 | 5 |  |
| Male sex | 103 (55) | 16 (53) | 1 | 33 (65) | 9 (56) | 0.57 | 39 (57) | 3 (33) | 0.29 | 31 (46) | 4 (80) | 0.19 |
| Parents primary caregiver | 168 (89) | 29 (97) | 0.32 | 51 (100) | 16 (100) | n/a | 62 (90) | 8 (89) | 1 | 55 (81) | 5 (100) | 0.58 |
| Year of ART initiation |  |  |  |  |  |  |  |  |  |  |  |  |
| 2008-2010 | 127 (68) | 15 (50) |  | 24 (47) | 6 (38) |  | 48 (70) | 4 (44) |  | 55 (81) | 5 (100) |  |
| 2011-2015 | 61 (32) | 15 (50) | 0.07 | 27 (53) | 10 (62) | 0.57 | 21 (30) | 5 (56) | 0.15 | 13 (19) | 0 | 0.58 |
| Underweight at ART | 84 (46) | 23 (50) | 0.68 | 13 (27) | 5 (42) | 0.31 | 35 (51) | 4 (44) | 0.74 | 36 (54) | 4 (80) | 0.37 |
| Severe immunodeficiency at ART | 87 (51) | 15 (58) | 0.53 | 18 (45) | 6 (50) | 1 | 38 (58) | 6 (67) | 0.73 | 31 (48) | 3 (60) | 0.67 |
| Log_10_ viral load at ART, median (IQR) | 5.7  (5.2, 5.9) | 5.4  (5.1, 5.9) | 0.76 | 5.9  (4.9, 6.3) | 5.6  (5.3, 5.9) | 0.86 | 5.9  (5.3, 5.9) | 5.9  (3.3, 5.9) | 0.85 | 5.5  (5.2, 5.9) | 5.1  (3.8, 5.6) | 0.26 |
| First regimen |  |  |  |  |  |  |  |  |  |  |  |  |
| AZT/3TC/EFV | 29 (15) | 2 (7) |  | 3 (6) | 0 (0) |  | 11 (16) | 2 (22) |  | 15 (22) | 0 (0) |  |
| AZT/3TC/NVP | 33 (18) | 9 (30) |  | 15 (29) | 5 (31) |  | 10 (14) | 3 (33) |  | 8 (12) | 1 (20) |  |
| D4T/3TC/EFV | 37 (20) | 2 (7) |  | 4 (8) | 1 (6) |  | 20 (29) | 1 (11) |  | 13 (19) | 0 (0) |  |
| D4T/3TC/NVP | 65 (35) | 13 (43) |  | 17 (33) | 6 (38) |  | 18 (26) | 3 (33) |  | 30 (44) | 4 (80) |  |
| Other | 24 (13) | 4 (13) | 0.16 | 12 (24) | 4 (25) | 1 | 10 (14) | 0 (0) | 0.39 | 2 (3) | 0 (0) | 0.44 |

ART: antiretroviral therapy; CI: confidence interval; n/a: not applicable; Ref: reference group

Note: viral suppression defined as two consecutive undetectable (<400 copies/mL) viral load measures after ART initiation

Underweight: Weight-for-age Z-score less than -2, Severe Immunodeficiency: Defined by 2006 WHO Guidelines [19]

3TC: Lamivudine, ART: antiretroviral therapy, AZT: Zidovudine, D4T: Stavudine, EFV: Efavirenz, NVP: Nevirapine

^1^ p-value for comparison of age groups from Pearson chi-square test

**Supplementary Table 9. Outcomes among children living with HIV and receiving treatment in rural Zambia, overall and stratified by age at ART initiation**

| **Outcome by the end of the study period** | **All ages** | **<1 year** | **1 to <2 years** | **2 to <5 years** |
| --- | --- | --- | --- | --- |
| **In care** |  |  |  |  |
| **N (%)** | 279 (73.2) | 60 (69.8) | 99 (66.7) | 123 (81.5) |
| **Median months of FU (IQR; range)** | 36 (36-36; 5-36) | 36 (35-36; 12-36) | 36 (36-36; 6-36) | 36 (36-36; 5-36) |
| **Transferred out** |  |  |  |  |
| **N (%)** | 39 (10.2) | 6 (7.0) | 20 (13.9) | 13 (8.6) |
| **Median months of FU (IQR; range)** | 18 (7-26; 1-36) | 17 (3-19; 3-31) | 18 (10-23; 3-36) | 17 (6-29; 1-33) |
| **Lost to follow-up** |  |  |  |  |
| **N (%)** | 14 (3.7) | 6 (7.0) | 4 (2.8) | 4 (2.7) |
| **Median months of FU (IQR; range)** | 14 (12-24; 3-29) | 17 (5-24; 3-28) | 19 (14-26; 13-29) | 12 (10-18; 8-24) |
| **Cumulative % at 36 months (95% CI)** | 4.6 (1.2, 7.8) | 9.3 (4.2, 20.7) | 3.7 (1.4, 9.8) | 3.1 (1.1, 8.1) |
| **Death** |  |  |  |  |
| **N (%)** | 49 (12.9) | 14 (16.3) | 24 (16.7) | 11 (7.3) |
| **Median months of FU (IQR; range)** | 4 (1-8; 0-29) | 3 (1-10; 0-21) | 4 (1-7; 0-29) | 3 (1-8; 0-25) |
| **Cumulative % at 36 months (95% CI)** | 15.2 (11.5, 20.2) | 21.0 (12.5, 35.5) | 19.6 (13.1, 29.3) | 8.3 (4.6, 14.9) |
| **Mortality rate per 1000 child-months (95% CI)** | 4.5 (3.4, 5.9) | 6.1 (3.6, 10.3) | 6.0 (4.1, 9.0) | 2.4 (1.3, 4.3) |

CI: confidence interval; FU: follow-up; IQR: interquartile range

**Supplementary Table 10. Risk factors for mortality among children living with HIV and receiving treatment in rural Zambia, overall and stratified by age at ART initiation**

|  | All ages | | <1 year | | 1 to <2 years | | 2 to <5 years | |
| --- | --- | --- | --- | --- | --- | --- | --- | --- |
| Characteristic at ART initiation | **Crude** | **Adjusted^1^** | **Crude** | **Adjusted^2^** | **Crude** | **Adjusted^2^** | **Crude** | **Adjusted^2^** |
|  | **Hazard Ratio (95% CI)** | **Hazard Ratio (95% CI)** | **Hazard Ratio (95% CI)** | **Hazard Ratio (95% CI)** | **Hazard Ratio (95% CI)** | **Hazard Ratio (95% CI)** | **Hazard Ratio (95% CI)** | **Hazard Ratio (95% CI)** |
| Age group |  |  | n/a | n/a | n/a | n/a | n/a | n/a |
| <1 year | 2.55 (1.16, 5.64) | 3.41 (1.40, 8.29) |  |  |  |  |  |  |
| 1 to <2 years | 2.36 (1.15, 4.81) | 2.53 (1.14, 5.59) |  |  |  |  |  |  |
| 2 to < 5 years | Ref | Ref |  |  |  |  |  |  |
| Female | 0.68 (0.39, 1.20) | 0.57 (0.30, 1.09) | 0.42 (0.15, 1.21) | 0.32 (0.08, 1.20) | 0.51 (0.22, 1.16) | 0.52 (0.21, 1.28) | 1.47 (0.45, 4.81) | 0.92 (0.23, 3.59) |
| Severe immunodeficiency | 1.68 (0.90, 3.13) | 1.43 (0.74, 2.76) | 2.08 (0.64, 6.78) | 2.81 (0.69, 11.50) | 1.21 (0.50, 2.92) | 1.00 (0.39, 2.56) | 2.26 (0.58, 8.75) | 1.95 (0.48, 7.85) |
| Underweight | 2.26 (1.24, 4.12) | 1.86 (0.98, 3.53) | 2.14 (0.72, 6.38) | 0.74 (0.17, 3.19) | 2.78 (1.10, 7.00) | 2.99 (1.09, 8.22) | 1.86 (0.54, 6.36) | 1.45 (0.37, 5.67) |
| Viral load (log_10_ copies/ml) | 1.18 (0.74, 1.88) | --- | 1.20 (0.65, 2.23) | --- | 1.54 (0.62, 3.79) | --- | 0.40 (0.14, 1.11) | --- |
| Year |  |  |  |  |  |  |  |  |
| 2008-2010 | Ref | Ref | Ref | Ref | Ref | Ref | Ref | Ref |
| 2011-2015 | 0.48 (0.26, 0.86) | 0.30 (0.12, 0.74) | 0.32 (0.10, 1.03) | 0.13 (0.02, 0.70) | 0.50 (0.22, 1.15) | 0.34 (0.09, 1.31) | 0.58 (0.17, 1.97) | 0.60 (0.06, 6.54) |
| Regimen |  |  |  |  |  |  |  |  |
| NNRTI/PI |  |  |  |  |  |  |  |  |
| NVP | Ref | --- | Ref | --- | Ref | --- | Ref | --- |
| EFV | 1.18 (0.65, 2.14) | --- | 3.79 (1.22, 11.78) | --- | 1.67 (0.51, 2.69) | --- | --- | --- |
| LPV/r | 0.39 (0.09, 1.66) | --- | 0.57 (0.07, 4.72) | --- | 0.30 (0.04, 2.36) | --- | --- | --- |
| NRTI |  |  |  |  |  |  |  |  |
| AZT | Ref |  | Ref | --- | Ref | --- | Ref | --- |
| D4T | 2.11 (1.06, 4.21) | 0.83 (0.35, 1.90) | 2.17 (0.56, 8.40) | 0.43 (0.07, 2.55) | 1.41 (0.57, 3.50) | 0.56 (0.17, 1.83) | 7.33 (0.90, 59.59) | 5.20 (0.48, 56.08) |
| ABC | 1.57 (0.66, 3.72) | 1.87 (0.73, 4.83) | 1.62 (0.33, 8.02) | 0.96 (0.18, 5.16) | 0.96 (0.29, 3.20) | 1.39 (0.34, 5.68) | 5.21 (0.47, 57.45) | 7.97 (0.55, 114.76) |

ABC: Abacavir, ART: antiretroviral therapy, AZT: Zidovudine, CI: confidence interval, D4T: Stavudine, EFV: Efavirenz, LPV/r: Lopinavir / Ritonavir, NVP: Nevirapine, NNRTI: non-nucleoside reverse transcriptase inhibitor, NRTI: nucleoside reverse transcriptase inhibitor, PI: protease inhibitor, Ref: reference group

Underweight: Weight-for-age Z-score less than -2, Severe immunodeficiency: Defined by 2006 WHO Guidelines [19]

Note: Due to missingness, the total N and events are not equal between the crude and adjusted model

^1^ Adjusted for age, sex, severe immunodeficiency, underweight, year, and NRTI backbone at ART initiation

^2^ Adjusted for sex, severe immunodeficiency, underweight, year, NRTI backbone at ART initiation
