# Supplementary figures and images for: Treatment outcomes among children younger than five years living with HIV in rural Zambia, 2008–2018: a cohort study
Source: BMC Pediatr. 2021 Jul 14;21:315. doi: 10.1186/s12887-021-02793-y (PMC8278691; doi:10.1186/s12887-021-02793-y)

a. < 1 year (n=67)

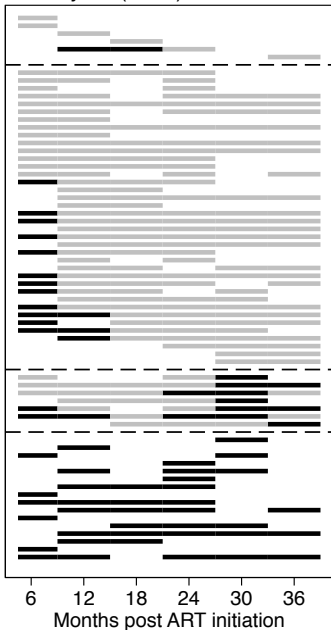

b. 1 year to < 2 years (n=78)

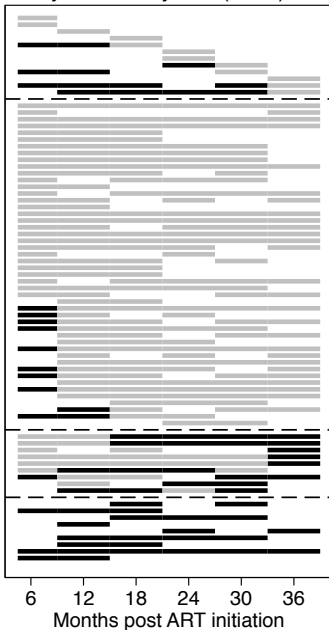

c. 2 years to < 5 years (n=73)

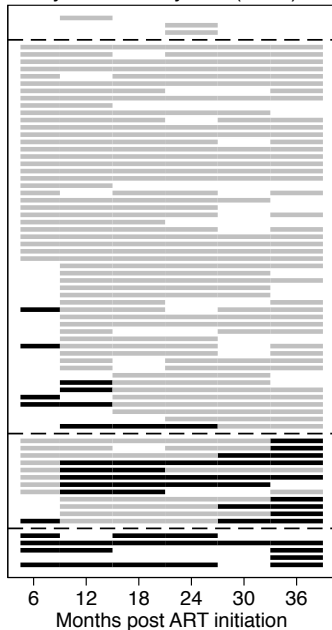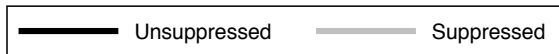

Supplement: Supplementary file 2 — Additional file 2: Supplementary Figure 1. Viral suppression among children living with HIV and receiving treatment in rural Zambia, 2008–2018. Note: Each row represents the experience of a participant. Viral suppression was defined as two consecutive viral loads < 400 copies per mL among available samples. (A)-(C): Children were grouped into 4 categories, each separated by a dashed line on the panel. Children in the 4th category never achieved viral suppression, the 3rd category reached suppression but did not maintain suppression, the 2nd category achieved and maintained viral suppression, and the 1st category reached suppression but did not have a follow up visit to measure maintenance. (A) The median (IQR) number of viral load measures available after ART initiation was 6 (5, 6). Forty-five of fifty-one children achieving viral suppression had at least one viral load measure available to assess maintenance of suppression. The median (IQR) number of additional viral load measures available was 2 (0, 3). (B) The median (IQR) number of viral load measures available after ART initiation was 6 (5, 6). Fifty-seven of sixty-nine children achieving viral suppression had at least one viral load measure available to assess maintenance of suppression. The median (IQR) number of additional viral load measures available was 2 (0, 4). (C) The median (IQR) number of viral load measures available after ART initiation was 5 (5, 6). Forty-five of fifty-one children achieving viral suppression had at least one viral load measure available to assess maintenance of suppression. The median (IQR) number of additional viral load measures available was 4 (3, 5). [file 12887_2021_2793_MOESM2_ESM.pdf]
