# Supplementary material for: Treatment outcomes among children younger than five years living with HIV in rural Zambia, 2008–2018: a cohort study
Source: BMC Pediatr. 2021 Jul 14;21:315. doi: 10.1186/s12887-021-02793-y (PMC8278691; doi:10.1186/s12887-021-02793-y)
Supplement: Supplementary file 3 — Additional file 3: Supplementary Figure 2. Loss to follow-up after treatment initiation among children living with HIV in rural Zambia, 2008–2018. [file 12887_2021_2793_MOESM3_ESM.pdf]

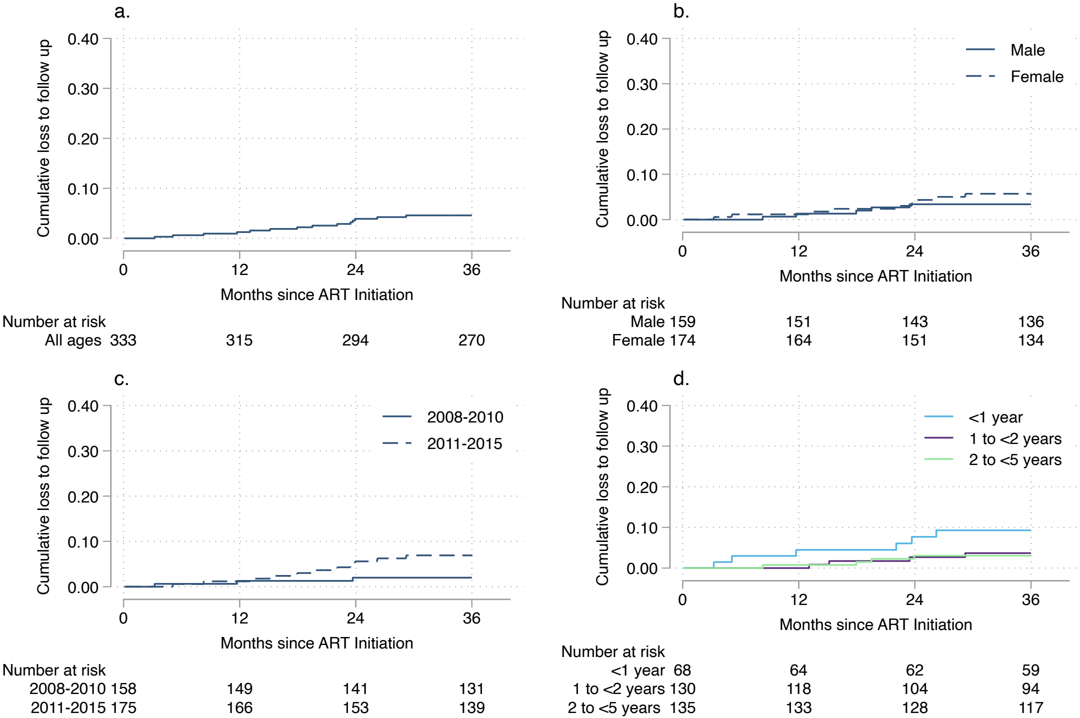

a.
Cumulative loss to follow up
0.40
0.30
0.20
0.10
0.00
0
12
24
36
Months since ART Initiation
Number at risk
All ages 333 315 294 270
b.
Male
Female
Cumulative loss to follow up
0.40
0.30
0.20
0.10
0.00
0
12
24
36
Months since ART Initiation
Number at risk
Male 159 151 143 136
Female 174 164 151 134
c.
2008-2010
2011-2015
Cumulative loss to follow up
0.40
0.30
0.20
0.10
0.00
0
12
24
36
Months since ART Initiation
Number at risk
2008-2010 158 149 141 131
2011-2015 175 166 153 139
d.
<1 year
1 to <2 years
2 to <5 years
Cumulative loss to follow up
0.40
0.30
0.20
0.10
0.00
0
12
24
36
Months since ART Initiation
Number at risk
<1 year 68 64 62 59
1 to <2 years 130 118 104 94
2 to <5 years 135 133 128 117
